# Measuring feelings of dehumanization in people who experience psychosis: development and validation of the self-Dehumanization in Psychosis Scale (DiPS)

**DOI:** 10.1093/schbul/sbaf242

**Published:** 2026-03-21

**Authors:** Tom A Jenkins, Pamela Jacobsen, Paul Chadwick

**Affiliations:** Department of Psychology, University of Bath, Bath, United Kingdom; Department of Psychology, University of Bath, Bath, United Kingdom; Department of Psychology, University of Bath, Bath, United Kingdom

**Keywords:** psychometric, otherness, schizophrenia, hallucinations, paranoia

## Abstract

**Background and Hypothesis:**

Self-dehumanization is the experience of feeling less or other than human, and is known to be experienced by people with psychosis. Existing measures of self-dehumanization are limited in their applicability to psychosis, and have not been developed with people with lived experience. The aim of this study was to develop and validate a measure of self-dehumanization in psychosis in partnership with key stakeholder groups.

**Study Design:**

Firstly, domains were specified based on review of existing theories of self-dehumanization and qualitative research on self-dehumanization in psychosis. Secondly, items were generated from a systematic literature review of existing measures of self-dehumanization, transcripts from qualitative research on self-dehumanization in psychosis, and lived experience consultations. Third, items were reduced and revised in a Delphi study (*n* = 49). Fourth, cognitive interviews (*n* = 9) were conducted to improve comprehensibility and further revise items. Finally, in psychometric validation, the DiPS underwent exploratory and confirmatory factor analysis, item reduction, and reliability and validity assessment (*n* = 456).

**Study Results:**

The 13-item DiPS was developed. Both two- and four-factor solutions were tested; the four-factor solution, comprising *Humanity*, *Identity*, *Personhood*, and *Agency*, demonstrated optimal fit. The DiPS showed strong construct validity, correlating positively with internalized stigma, paranoid thoughts, and voice-hearing, and negatively with self-compassion. Test–retest reliability and internal consistency were excellent.

**Conclusions:**

The DiPS is a reliable and valid measure of self-dehumanization in psychosis. This novel measure can be used in research and clinical practice to better understand distress in psychosis.

## Introduction

Psychological essentialism is the belief that things have both a physical form—a “kind”—and an invisible, inherent quality that determines their characteristics as a member of that kind—an “essence.”^1^^,^^2^ Dehumanization is when a person or group are perceived to lack the human essence, and is often a precursor to violence, abuse, and other inhumane treatment.^3-5^ Self-dehumanization is when a person feels less than or other than human: it is the felt sense of being human in form, but not in essence. Self-dehumanization shares conceptual overlap with other self and identity-related experiences, including internalized stigma, low self-esteem, self-objectification, and depersonalization/derealization. Although these experiences may co-occur,^6-8^ they do not all refer to a perceived absence of humanness. Self-dehumanization extends beyond perceptions of self-worth and embodiment to manifest as a deep sense of otherness and diminished humanity.

Self-dehumanization can be an especially challenging and distressing experience for people who experience psychosis. Meta-dehumanizationis the perception of being seen or treated as less human, and is moderately associated with self-dehumanization.^9^ People with psychosis are often seen as less human^10^^,^^11^ and may have been subject to dehumanizing experiences, such as trauma,^12^^,^^13^ racism,^14^^,^^15^ social isolation,^16^^,^^17^ and involuntary hospitalization or medication^18^—all of which could precipitate self-dehumanization. Furthermore, the experience of psychosis can be inherently dehumanizing. In the first qualitative study to explore experiences of self-dehumanization, O’Brien-Venus, Jenkins, and Chadwick^19^ interviewed 20 people with experience of distressing voice-hearing, developing a theoretical model of self-dehumanization in psychosis. Self-dehumanization was positioned as the end of an experiential continua constituted by distressing sensory fragmentation from voices, a lost sense of belonging with other humans, erosion of trust in personal credibility and reliability, lower sense of worth as a human, reduced strength of personal agency, and a disintegration of the self as a private and coherent entity. Self-dehumanization occurred in the experience of one or more facets of the continua. Treatment by others, in a humanizing or dehumanizing (meta-dehumanization) manner, drew participants towards or away from self-dehumanization. O’Brien-Venus, Jenkins, and Chadwick’s study builds on contemporary social psychology conceptualizations, which define self-dehumanization as the denial of human nature (e.g. civility, morality, rationality) and/or human uniqueness (e.g. warmth, emotionality, individuality) attributes,^20^ or mental capacities (e.g. emotion, intention, and cognition)^21^ to the self. O’Brien-Venus and colleagues capture the dynamic and existential dimensions of self-dehumanization in psychosis, providing a phenomenological account that serves as the focal point for understanding experiences of dehumanization in relation to psychosis.

Self-dehumanization is recognized as a possible treatment target for mental health interventions,^22^ with evidence to suggest it is associated with anxiety, depression, and concerningly, suicidal ideation and intent.^8^^,^^9^^,^^23^^,^^24^ Given the emerging evidence of self-dehumanization in psychosis, it is important to understand how the experience manifests and how people who feel dehumanized can be effectively supported. To enable this, a measure of self-dehumanization in psychosis must be developed and validated. Existing self-dehumanization measures^8^^,^^9^^,^^20-22^ do not have input from people with lived experience—a key aspect of content validity^25^—and have not been developed for a psychosis population, therefore may not account for the uniquely dehumanizing aspects of psychosis symptoms.^19^ Measures developed in collaboration with people with lived experience of mental health conditions are deemed both more relevant and more acceptable to those who complete them.^26^

The present study therefore has the following aims: (1) develop a measure of self-dehumanization in psychosis, involving people with lived experience and other expert stakeholders, and (2) validate the measure in a clinical sample of people with psychosis. It was hypothesized that self-Dehumanization in Psychosis Scale (DiPS) scores will positively correlate with internalized stigma and voice-hearing, given existing associations between these variables;^5^^,^^19^ paranoid thoughts, as negative self-schema and social defeat are implicated in paranoia;^27^^,^^28^ and negatively correlate with self-compassion, as self-dehumanization represents the inverse of the “common humanity” component of Neff’s model.^29^

## Methods

### Design

Measure development and validation followed best practice recommendations from Boateng, Neilands, Frongillo, Melgar–Quiñonez, and Young:^30^ (1) The construct and domains were identified through a review of theories of self-dehumanization, pre-existing qualitative research on self-dehumanization and psychosis,^19^ and a Patient and Public Involvement and Engagement (PPIE) focus group consultation. (2) Items were generated from a systematic literature review of existing measures of self-dehumanization, pre-existing qualitative study transcripts,^19^ research team discussions, and focus groups with PPIE consultants. (3) Items were refined via a Delphi study with different expert stakeholder groups. (4) Pre-testing of candidate items took place with cognitive “think aloud” interviews with people with experience of psychosis. (5) Psychometric validation reduced items and determined factor structure, reliability, and validity.

### Ethical Approval

Stages 1-4 did not recruit from NHS clinical services so was approved by the University of Bath Psychology Research Ethics Committee (Ref: 22 157). Stage 5, DiPS Validation (IRAS ID: 333807) received HRA and Health and Care Research Wales approval by South West – Cornwall & Plymouth Research Ethics Committee (23/SW/0150).

### DiPS Lived Experience Consultation Group

The DiPS lived experience consultation group comprised five people with personal experience of psychosis and mental health service use. They were recruited via a local newsletter sent to the People with Personal Experience Committee at the University of Bath. They will be referred to throughout as “PPIE consultants”, to reflect their input as research consultants throughout the study. PPIE consultants contributed to the study through two focus groups informing item generation, review, and revision; item reduction following exploratory factor analysis; providing feedback on participant facing documents; and supporting with dissemination. Members of the group were reimbursed £25/hour for their involvement, in line with National Institute of Health Research (NIHR) guidelines.^31^

### Defining Self-Dehumanization and Domain Specification

O’Brien-Venus, Jenkins, and Chadwick’s^19^ theoretical model of self-dehumanization and a review of existing dehumanization theory^4^^,^^20^^,^^21^ was used to represent domains of self-dehumanization. This was presented to PPIE consultants in Focus Group 1, who were consulted for feedback on the validity for this representation of self-dehumanization and the relevance of conducting this research. Pragmatic decisions were made to ensure all domains of the theoretical model were adequately represented throughout each stage of measure development.

### Item Generation

Items were generated both deductively and inductively, with the research team (which comprised one PhD student and two clinical academic psychologists, all of whom had experience of working with people with psychosis) meeting regularly to discuss candidate items throughout the process. The process was as follows:

#### Deductive Item Generation

A systematic search of Scopus, PubMed, Web of Science, and APA PsycNet was conducted on 13 December 2022 using the terms “self dehumanization” and “self infrahumanization,” with the aim of retrieving existing peer-reviewed self-report measures of self-dehumanization across all publication years (see supplementary materials for search strategy). The research team reviewed all items and only those directly measuring self-dehumanization were included in the shortlist for the Delphi study.

#### Inductive Item Generation

Items were developed from quotes and thematic summaries from O’Brien-Venus, Jenkins, and Chadwick’s qualitative study on experiences of self-dehumanization in voice-hearers,^19^ and PPIE consultants in Focus Group 1. The research team met to review and revise these items, adding further items based on experience from clinical practice. A second focus group with PPIE consultants then took place to review all items and suggest further items. The research team conducted a final review to integrate these suggestions and finalize the shortlist for the Delphi study.

### Delphi Study

#### Participants

Four stakeholder groups took part in the Delphi: (1) People with experience of distressing psychosis, defined as voice-hearing, hallucinations (in any modality), delusions, or paranoia; (2) mental health professionals: anyone with current or recent employment providing psychological support to people with psychosis; (3) dehumanization researchers: anyone who has published at least one academic paper on dehumanization; (4) carers of people with psychosis: anyone providing unpaid care to a person with psychosis.

All participants were required to be 18 years or above and able to comprehend English well enough to understand the study materials. Participants with experience of psychosis and carers were recruited via adverts placed on Twitter, MQ Participate, Mind, the Carers Center, University of Bath, and word of mouth. Eligible researchers and mental health professionals from professional networks were contacted directly. People with experience of psychosis and carers were reimbursed with a £15 voucher. Researchers and mental health professionals were not reimbursed as their involvement was undertaken within the scope of their regular paid work.

#### Procedure

A modified 2-round Delphi method was used. Participants reviewed seven item sets, each corresponding to a proposed domain of self-dehumanization, and rated the importance of each item for measuring its respective domain on a 9-point Likert scale (1 = “Not important,” 9 = “Critical”). In Round 1 only, participants could provide qualitative feedback for each item, overall feedback at the end of the study, and suggest new items. In Round 2, participants re-rated items which did not reach consensus, and those which had been newly suggested in Round 1. Both rounds were open for seven weeks.

#### Analysis and Materials

Consensus limits for Round 1 were based on previous Delphi studies in psychosis research,^32^^,^^33^ defined as:

1. If at least 80% of stakeholders in all four groups rate an item as “critical,” it is included
2. If 80% or more of stakeholders from one, two, or three of the groups rates an item as critical, all stakeholders are asked to rerate that item
3. If 70%-79% of stakeholders from all four groups rate an item as critical, all stakeholders are asked to rerate that item
4. Any items that do not meet the above three conditions will be excluded

Following analysis of Round 1 data, these limits were deemed to be overly restrictive, as no items met the threshold for automatic inclusion. Consensus limits were modified for Round 2 and defined as:

1. If at least 66.66% of stakeholders across three or more groups rate an item as “critical,” it is included
2. If at least 66.66% of *people with psychosis* rate an item as “critical” in either round 1 OR 2, it is included
3. If neither condition 1 or 2 are satisfied, the item is excluded

Data was collected using DelphiManager (COMET initiative). Data was analyzed using R Studio, version 4.3.1^34^ and Microsoft Excel.

### Cognitive Interviews

#### Participants

People with experience of distressing psychosis, defined as voice-hearing, hallucinations (in any modality), delusions, or paranoia were recruited. All participants were required to be 18 years or above and able to comprehend English well enough to understand the study materials. Recruitment methods were the same as in Stage 3. Participants were reimbursed with a £10 voucher.

#### Procedure

Participants read each item of the DiPS and invited to “think aloud,” articulating their thought process as if they were intending to answer the question.^35^ The interviewer would occasionally use verbal probes to ask specifically what the participant perceived particular words within certain items to mean. Interviews took place online via Microsoft Teams. Data were analyzed with Microsoft Excel.

#### Analysis

The interviewer made notes on participant responses as they answered and reported the main themes of feedback for each item. Analysis involved a consideration of the implications of each participant’s response. Items were amended or discarded based on the following self-set criteria:

- If an item caused offense to one participant, it would be amended.

- If an item was misinterpreted by two or more participants, it was amended.

- If an item was deemed a repetition of a previous item by two or more participants, it was discarded.

Interviews took place in rounds of three participants and data were analyzed via an iterative process. After three participants completed the interviews, data were analyzed, and amendments made based on responses. This continued until a point where a round of three participants reported no issues with item comprehension.

### Psychometric Validation

#### Participants

Participants were recruited from both NHS services (*n* = 383) and non-NHS routes (*n* = 73) between January 29 2024 and February 12 2025. NHS recruitment took place across 16 sites in England and Wales with support from the NIHR Clinical Research Network. Non-NHS recruitment included referrals of potentially eligible participants from the STOP trial (ISRCTN17754650); MQ Participate advertisement; and mail-outs sent by NIHR Be Part of Research, McPin Foundation, and National Survivor User Network.

Participants recruited from NHS services were eligible if they had either a diagnosis of schizophrenia-spectrum disorder (F20-F29 on ICD10) or eligibility for Early Intervention in Psychosis services. Those recruited from non-NHS routes were screened in a phone call by the lead researcher in which they were assessed for eligibility using the PSYRATS.^36^ Participants were eligible if reporting the presence of moderately distressing hallucinations or delusions on at least a weekly basis. All participants were required to be 18 years or older, and able to understand English well enough to comprehend the study materials. Participants were incentivized with the chance to enter a prize draw for a £50, £20, or £10 shopping voucher.

### Measures

#### self-Dehumanization in Psychosis Scale (DiPS)

The DiPS was presented as a 22-item self-report questionnaire measuring self-dehumanization over the past week, with items generated from the measure development phase outlined in this paper. Likert scale response options range from 0 (never) to 4 (always). Higher scores indicate greater self-dehumanization. The final 13-item version of the scale is included in the appendix, and the 22-item version is included in Supplementary Materials.

#### *Internalized Stigma of Mental Illness Inventory-9 (ISMI-9)* *^37^*

The ISMI-9 is a 9-item self-report questionnaire measuring internalized stigma. It was adapted for the purpose of this study, replacing the word “mental illness” in each item with “psychosis.” Likert-scale response options range from 1 (strongly disagree) to 4 (strongly agree). Higher scores indicate greater internalized stigma. α = .86.

#### *Self-Compassion Scale Short Form (SCS-SF)* *^38^*

The SCS-SF is a 12-item self-report questionnaire measuring self-compassion. Likert scale response options range from 1 (almost never) to 5 (almost always). Higher scores indicate greater self-compassion. α = .85.

#### *Revised Green*  *et al.*  *Paranoid Thoughts Scale (R-GTPS)**^39^*

The R-GPTS is an 18-item self-report questionnaire measuring paranoid thoughts over the past month. It contains two subscales: ideas of reference, and ideas of persecution. Likert scale response options range from 0 (not at all) to 4 (totally). Higher scores indicate more paranoid thoughts. α = .96.

#### *Hamilton Program for Schizophrenia Voices Questionnaire (HPSVQ)* *^40^*

The HPSVQ is a 13-item self-report questionnaire measuring characteristics and impact of voice-hearing over the past week. It was selected for its suitability as a self-administered measure, and demonstrates some evidence of internal consistency; test–retest reliability; convergent, structural, and criterion validity.^40^^,^^41^ It was adapted for the purpose of this study, with only items 1-9 administered (items 10-13 form a qualitative assessment of voice-hearing). Likert scale response options range from 0 (least severe or impairing) to 4 (most severe or impairing). Higher scores indicate greater severity and impairment of voice-hearing. α = .96.

### Procedure

A cross-sectional study design was used. Participants gave informed consent, provided demographic information, and completed all measures. Three days after completing, a subset of consenting participants were followed up with a request to complete the DiPS once more within the following four days for test–retest reliability. Participants completed the questionnaires either online via REDCap^42^^,^^43^ hosted by the University of Bath, or on a paper copy. For the online questionnaires, all items were mandated, resulting in no missing data. For the paper copies, questionnaires returned with <20% missing items had missing data replaced by the mean of the questionnaire score. Any paper copies returned with missing questionnaires (*n* = 3) were excluded from the analysis.

### Analysis

Analysis was conducted in R Studio, version 4.3.1.^34^ Data were collected, stored, and managed on REDCap.^42^^,^^43^

Data were split into exploratory factor analysis (EFA) (*n* = 220) and confirmatory factor analysis (CFA) (*n* = 236) samples. Sample size for factor analysis was guided by Boateng and colleagues recommendation^30^ for at least *n* = 10 participants per number of items, and a target of *n* = 220 was set for both EFA and CFA samples. Slight over-recruitment occurred for the CFA sample, hence analysis was conducted on *n* = 236. In the EFA sample, inter-item correlations were performed on the DiPS to determine item redundancy. EFA was conducted iteratively, using differing combinations of items based on factor loadings (>.30), cross-loadings, multicollinearity (>.80) and non-collinearity (<.30). Items which violated any of these criteria were considered by the research team and PPIE consultants for removal. A subsequent EFA with oblique rotation was performed on the remaining items; number of factors was based on consideration of the scree plot, parallel analysis, and Kaisers’ criterion. CFA was then carried out on the CFA sample using the maximum likelihood estimator. COSMIN^25^ guidance for good model fit was followed, which recommends Tucker Lewis Index (TLI) and Comparative Fit Index (CFI) >0.95, Root Mean Squared Error of Approximation (RMSEA) <0.06, and Standardized Root Mean Squared Residual (SRMR) <0.08.

Internal consistency was measured using Cronbach’s alpha for total and subscales. Adjusted item-total correlations were examined to determine whether any remaining items had low correlation with total scale score. Test–retest reliability was determined for a subset of n = 197 using a Pearson’s correlation between DiPS scores at timepoint 1 and timepoint 2 (3-7 days later). Although this relatively brief period increased the likelihood of recall bias, it was selected to reduce the likelihood of psychosocial factors influencing self-dehumanization scores over a longer period. Floor and ceiling effects were tested for by determining if more than 15% of participants scored the minimum or maximum score on the DiPS.^44^ Correlations between DiPS scores and internalized stigma (ISMI-9), self-compassion (SCS-SF), voices (HPSVQ), and paranoid thoughts (R-GPTS) were investigated to determine construct validity.

## Results

### Defining Self-Dehumanization and Domain Specification

Seven domains of self-dehumanization were identified: six derived from O’Brien-Venus, Jenkins, and Chadwick’s^19^ qualitative study on self-dehumanization in voice-hearers, and one from a review of existing theory.^4^^,^^20^^,^^21^ PPIE consultants in Focus Group 1 agreed the domains accurately reflected the experience of self-dehumanization. The final domains were: *Dehumanization*; *Extent of distressing sensory fragmentation; Sense of belonging with other humans*; *Integrity of self as a private, coherent entity; Sense of worth as a human being*; *Trust in own credibility and reliability*; *Strength of personal agency*.

### Item Generation

#### Deductive Item Generation

The systematic review identified eleven measures of self-dehumanization (See Supplementary Materials Table 1).^6^^,^^7^^,^^20^^,^^21^^,^^23^^,^^45-47^ Six measures^6^^,^^7^^,^^20^^,^^21^^,^^23^ contained items in a suitable self-report Likert scale format. Of the 69 items in these six measures, only three items were suitable for inclusion. Reasons for exclusion were: measuring a construct deemed as not self-dehumanization (*n* = 24); measuring attributes, rather than experience (*n* = 18); ambiguous wording (*n* = 13); substantiality overlapping with, or the same as another item (*n* = 9); containing stigmatizing language or assumptions (*n* = 2). See Supplementary Materials for further information.

#### Inductive Item Generation

66 items were developed from the qualitative interview transcripts (*n* = 36) and Focus Group 1 (*n* = 30). These were reviewed by the research team, who removed 44 deemed to not be measuring self-dehumanization, and suggested a further 19. These 41 items, along with three from the deductive item generation were reviewed by PPIE consultants in a second focus group, who suggested 32 new items, removed 1, and reworded 8. The research team then reviewed all items and removed 16 deemed to be not measuring self-dehumanization, finalizing a 59 item shortlist for the Delphi study.

### Delphi Study

49 stakeholders took part in Round 1: 17 people with experience of psychosis, 15 mental health professionals, eleven dehumanization researchers, and six carers. 42 stakeholders took part in Round 2: 14 people with experience of psychosis, twelve mental health professionals, ten dehumanization researchers, and six carers. One mental health professional also had personal experience of psychosis. Further demographics are presented in Table 1.

**Table 1:** Participant Demographic Information

		**Delphi R1**	**Delphi R2**	**Cognitive interviews**	**Psychometric validation**
Variable		N (%)	N (%)	N (%)	N (%)
Age	18-24			2 (22.2)	45 (9.9)
	25-34			5 (55.5)	92 (20.2)
	35-44			1 (11.1)	122 (26.8)
	45-54			1 (11.1)	100 (21.9)
	55-64			0 (0)	81 (17.8)
	65+			0 (0)	16 (3.5)
Gender	Male	11 (22.4)	9 (21.4)	6 (66.6)	261 (57.2)
	Female	37 (75.5)	32 (76.2)	3 (33.3)	188 (41.2)
	Non-binary	1 (2)	1 (2.4)		5 (1.1)
	Prefer not to say				2 (0.4)
Ethnicity	Asian, Asian British, or Asian Welsh	3 (6.1)	3 (7.1)	1 (11.1)	26 (5.7)
	Black, Black British, Black Welsh, Caribbean or African	2 (4.1)	2 (4.8)	2 (22.2)	22 (4.8)
	Mixed or multiple ethnic groups	2 (4.1)	0 (0)	1 (11.1)	33 (7.2)
	White	42 (85.7)	37 (88.1)	5 (55.5)	368 (80.7)
	Other ethnic group			0 (0)	7 (1.5)
Diagnosis	Schizophrenia (F20)				209 (45.8)
	Delusional disorder (F22)				8 (1.8)
	Psychotic disorder (F23)				87 (19.1)
	Schizoaffective disorder (F25)				55 (12.1)
	Bipolar affective disorder (F31)				17 (3.7)
	Other				65 (14.3)
	None				15 (3.3)
Antipsychotic medication	Yes				400 (87.7)
	No				56 (12.3)

#### Round 1

**Figure 1:**
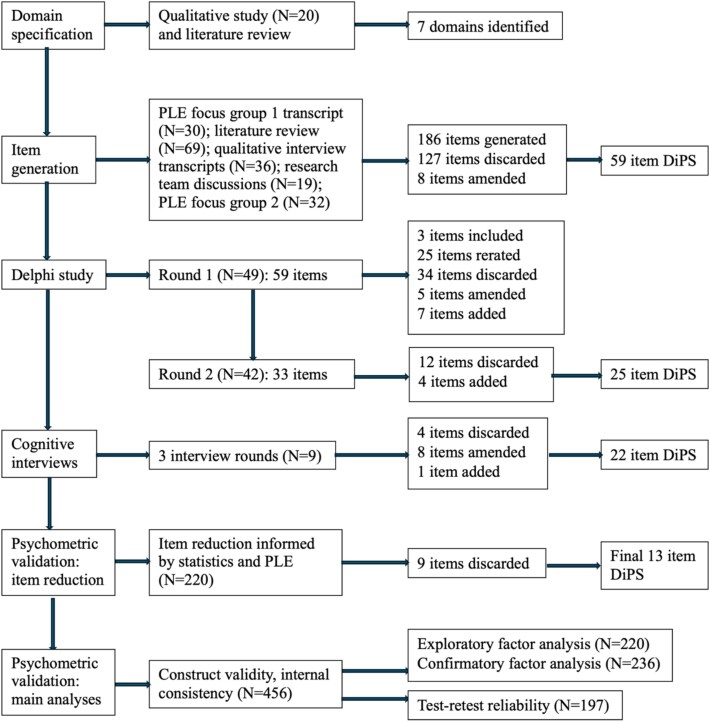
Flow of DiPS items across each stage of development and validation.

59 items were reviewed. None reached threshold for automatic inclusion. 20 items were put forward to be re-rated in Round 2. 5 items were amended and put forward for re-rating, based upon feedback provided by stakeholders. 34 items were excluded. Stakeholders suggested 22 new items, of which seven were included for rating in Round 2. Figure 1 displays the flow of items across each stage.

#### Round 2

33 items were reviewed. 20 items were included. Three items were included after being excluded from Round 1, given that more than 66.66% of people with experience of psychosis had previously rated them as critical in Round 1. Twelve items were excluded. Additionally, one item was reintroduced after completion of Round 2 to improve content validity and ensure comprehensive coverage of the conceptual domains.

### Cognitive Interviews

Nine people with experience of psychosis participated across three rounds. Participant demographics are displayed in Table 1. In Round 1, two items were removed, one due to ambiguity and the other judged to be repetition of others in the measure. Five statements were reworded, four of which due to ambiguity, and one of which caused offense to one participant. Based on participant feedback that two items were perceived as overlapping, an additional item was introduced to strengthen content validity and ensure comprehensive representation of the domain. In Round 2, three items were reworded due to ambiguity. In Round 3, two items were removed due to repetition. A full summary of changes can be found in Supplementary Materials Table 2. After three rounds of feedback and no new changes suggested, recruitment for the interview ended.

### Psychometric Validation

456 participants completed psychometric validation. Demographic information is presented in Table 1.

#### Item Reduction and Exploratory Factor Analysis

Item reduction from the original 22-item DiPS took place via iterative process which considered: theoretical coherence, factor cross-loadings, factor loadings (>.30), multicollinearity (*r* > .80), non-collinearity (*r* < .30), and PPIE consultant perspectives. Following a preliminary statistical analysis, twelve candidate items were considered for removal based on the aforementioned criteria, and presented to four members of the DiPS lived experience consultation group and two members of the research team who were asked to consider the relevance of each item along with overall content validity of the measure. It was deemed three should be retained, and nine should be removed. A further exploratory factor analysis (EFA) was then conducted on the 13 remaining items.

Sampling adequacy, assessed by the Kaiser–Meyer–Olkin (KMO) measure was “marvelous” (overall KMO = .90 all items KMO $\ge$ .83). Barlett’s test of sphericity indicated that correlations between variables were sufficient for EFA (χ^2^ (78) = 1371, 438, *P* < .001).

Kaiser’s criterion suggested the retention of two factors with eigenvalues greater than one. Scree plot suggested the retention of four factors. Parallel analysis suggested the retention of four factors (see Supplementary Materials Figures 1 and 2). After testing both two and four factor solutions, a four-factor structure was selected based on theoretical coherence, explaining 58% of the variance. Rotated factor loadings are displayed in Table 2. Individual factor loadings and communalities can be found in Supplementary Materials Table 3.

**Table 2:** Exploratory and Confirmatory Factor Loadings for the DiPS

**No.**	**Item**	**EFA**	**CFA**
Factor 1: Humanity			
7	I do not feel like a human being	.84	.68
11	I see myself as less than human	.84	.82
4	Distressing thoughts and voices make me feel like I am not human	.51	.71
10	I do not belong in this society	.46	.84
Factor 2: Identity			
5	Psychosis has taken away who I really am	.85	.76
12	Psychosis has taken over who I am	.82	.87
1	Psychosis prevents me from relating to other people	.37	.73
Factor 3: Personhood			
9	I have a strong sense of who I am as a person	.89	.80
3	I am a valuable person	.55	.68
13	I am more than my experience of psychosis	.44	.49
Factor 4: Agency			
6	I can’t trust myself to make good decisions	.58	.69
8	I can’t trust my mind	.48	.76
2	I have no control over my actions	.37	.54

The first factor, “*Humanity*” comprised four items capturing a feeling of not belonging to the human race. The second factor, “*Identity*” comprised three items relating to the experience of losing sense of self and connection to others. The third factor, “*Personhood*” comprised the three positively phrased items, conveying feelings of self-acceptance, worth, and knowledge. The fourth factor, “*Agency*” comprised items which conveyed a loss in autonomy and trust in oneself.

#### Confirmatory Factor Analysis

Confirmatory Factor Analysis (CFA) was conducted on the 13-item, four factor DiPS in the CFA sample. Excellent model fit was demonstrated: χ^2^(59) = 105.47, *P* < .001, CFI = 0.965, TLI = 0.953, RMSEA = 0.058, SRMR = 0.050. Table 2 and Supplementary Materials Figure 3 display standardized factor loadings.

#### DiPS (13 Item)

Descriptive statistics and correlations between all variables are displayed in Table 3.

**Table 3:** Descriptive Statistics for and Correlations between Each Variable in the DiPS Validation Study

**Variable**	**N**	**Scale range**	**Score range**	**Mean (SD)**	**Skewness**	**Kurtosis**	**DiPS**	**SCS-SF**	**ISMI-9**	**R-GPTS**	**HPSVQ**
DiPS (13 item)	456	0-52	0-49	22.16 (11.13)	−.01	−.79		−.67	.75	.61	.54
SCS-SF	456	12-60	12-60	33.63 (9.70)	.22	−.29		–	−.63	−.54	−.34
ISMI-9	456	9-36	9-36	22.20 (5.90)	−.18	−.25			–	.58	.41
R-GPTS	456	0-72	0-72	32.6 (21.57)	−.09	−1.18				–	.47
HPSVQ	456	0-36	0-36	12.80 (11.52)	.20	−1.42					–
HPSVQ (VH)^a^	293	1-36	1-36	19.91 (8.04)	−.19	−.83	.43	−.22	.37	.36	

All *P*<.001. DiPS, self-Dehumanization in Psychosis Scale; SCS-SF, Self Compassion Scale Short Form; ISMI-9, Internalized Stigma of Mental Illness Inventory-9; R-GPTS, Revised Green et al. Paranoid Thoughts Scale; HPSVQ, Hamilton Program for Schizophrenia Voices Questionnaire; HPSVQ (VH), Hamilton Program for Schizophrenia Voices Questionnaire (voice-hearers only).

a HPSVQ (VH) refers to a subset of only those participants with experience of voice-hearing, expressed by a score of 1 or more on the HPSVQ.

#### Internal Consistency

Internal consistency was assessed using Cronbach’s alpha statistic. Items on the DiPS demonstrated excellent internal consistency, with total α = .89. Alpha scores for each subscale were Humanity: α = .85, Identity: α = .83, Personhood: α = .70, Agency: α = .71. All items demonstrated adjusted item-total scores of *r* > .30.

#### Test–Retest Reliability

Pearson’s correlation between T1 and T2 for *n* = 197 participants showed a significant strong relationship, indicating excellent test–retest reliability: *r* (195) = .88 (CI = 0.84 to 0.91, *P* <.001).

#### Construct Validity

As hypothesized the DiPS showed significant positive correlations with R-GPTS (paranoia) (*r* [454] = .61), HPSVQ (voice-hearing) (*r* [454] = .54), and ISMI-9 (internalized stigma) (*r* [454] = .75). Also as hypothesized, DiPS showed significant negative correlation with the SCS-SF (self-compassion) (*r* [454] = −.67) (all *P* < .001). When those who scored 0 on the HPSVQ measure for voice-hearing (i.e. those who do not hear voices) were removed, there was a slight reduction in the strength of the correlation between the DiPS and the HPSVQ (*r* [291] = .43 [*P* < .001]). Table 3 displays descriptive statistics and correlations between each variable.

#### Floor and Ceiling Effects

Six participants scored the minimum of 0, and no participants scored the maximum of 52. This indicates that floor and ceiling effects are not present, in accordance with recommendations that no more than 15% of participants within a sample should register minimum or maximum scores.^44^

## Discussion

This study reports the development and validation of a novel measure of self-dehumanization in psychosis. Candidate items were generated from a literature review, qualitative interview transcripts,^19^ PPIE consultations, and members of the research team. These were presented to a group of expert stakeholders in a Delphi study, who voted on the most important items, along with suggesting new items. This list was further refined and amendments to item wording were made in cognitive interviews. Finally, psychometric analyses found the DiPS is both a reliable and valid measure of self-dehumanization in psychosis, comprising domains of *Humanity*, *Identity*, *Personhood*, and *Agency*.

The DiPS is the first self-dehumanization measure to draw on lived experience accounts in its development.^48^ Existing measures conceptualize self-dehumanization by either measuring humanness attributes (e.g. “I felt like I was refined and cultured”),^20^ mind attributions (e.g. “I can experience pleasure”),^21^ likening to non-human entities (e.g. “I am a monster”),^8^ or the emotional consequences of self-dehumanization (e.g. “I am disgusted by my lack of humanness”).^8^ Although only three of 69 possible items from the literature review were included in the initial item shortlisting, they were all rejected by people with experience of psychosis in the Delphi study. The DiPS contains language which is both acceptable and accessible to people who experience psychosis; accounts for uniquely dehumanizing aspects of psychosis^19^; and holds a unique factor structure capturing the experiential qualities of humanness. *Humanity* taps directly into the notion of feeling less or different to human, reflecting that one’s sense of humanity is connected to a feeling of belonging with other humans.^19^^,^^20^  *Personhood* comprises positively phrased items as suggested by PPIE consultants in focus groups, reflecting that to recognize and accept the inherent value of oneself is to feel more human. *Identity* reflects the importance of a balanced and dynamic sense of self^49^—accounts from people with lived experience describe feeling dehumanized by the disintegration of self in psychosis.^19^ A sense of personal *agency* is foundational to the human experience, aligning with psychological accounts of self-dehumanization,^20^^,^^21^ while recognized as a key mechanism for recovery in psychosis.^50^

This is the first study to investigate psychotic symptoms in relation to self-dehumanization, reporting strong correlations between voice-hearing, paranoid thoughts and self-dehumanization. This supports the idea posited by O’Brien-Venus, Jenkins, and Chadwick which suggests a collection of essentially human experiential continua (distressing sensory fragmentation, sense of belonging, agency, integrity of self, trust in oneself, and sense of worth) coalesce at the point of self-dehumanization.^19^ For positive symptoms, self-dehumanization is the point of extreme distress, and for negative symptoms, the point of extreme disconnection. A parallel can be found in phenomenological accounts of psychosis which suggest hyper reflexivity—a heightened form of self-consciousness in which ordinary aspects of experience are perceived as alien—can, in its most extreme manifestation, erode a person's sense of humanity.^51^^,^^52^

Self-dehumanization was related to internalized stigma, and inversely related to self-compassion, further demonstrating construct validity of the DiPS. This aligns with research on self-stigma and self-dehumanization in alcohol use disorder,^6^ and the idea that recognition of suffering as a universal human experience is a key constituent of self-compassion.^29^ The high correlation between the self-dehumanization and internalized stigma was as predicted. Internalized stigma is the loss of self-esteem and self-efficacy when an person adopts negative societal beliefs about their condition into their self-concept.^53^ Self-dehumanization, while also a negative self-perception, involves the collapse of this self-concept—it reflects a person’s sense that they no longer embody the essence of being human.

The DiPS can be used an outcome measure in both research and clinical practice across a variety of therapeutic modalities in addition to symptom measures and other key outcomes. Person-centered care is a key tenant of NHS service provision for psychosis. Therapies such as Cognitive Behavioral Therapy for psychosis (CBT-p) which emphasize a collaborative, caring therapeutic relationship as a vehicle for personal choice and recovery^54^ is much aligned with re-establishing one’s sense of humanness. Seeing oneself as less than human may also be a “core belief” which can be targeted by CBTp—indeed the idea of a “human essence” is suggested to be a cognitive bias.^1^ The development of “personhood” is an established component of humanistic “person based” therapy,^55^ and psychodynamic psychotherapy, which aims to help people with psychosis “recover confidence in their humanness”.^56^ Compassion Focused Therapy involves cultivating a sense of friendliness to oneself and has been shown to successfully target social rank process including self-criticism and shame in psychosis.^57^ A self-compassionate mindset is associated with less psychotic symptom distress^58^ and could be a promising therapeutic intervention given the association between self-dehumanization and self-compassion. Chadwick highlights the humanizing therapeutic benefits in group-based mindfulness for psychosis.^49^ Mindfulness for psychosis invites people to step back, let go of habitual reactions, and accept thoughts, voices, images, and feelings as transient experiences which do not define the self—supporting people to be more self-accepting and reclaim agency over their internal experience and daily life.^59^ PPIE consultants in the focus groups identified peer support, normalizing of psychotic experiences, and acceptance as humanizing processes—all of which are available in therapeutic groups with and without mindfulness practice. Group work for psychosis is known to give rise to sense of universality amongst clients—a recognition of the common humanity shared by all.^60^ The DiPS may also be useful in formulation; talking through the measure could facilitate a greater understanding for both client and clinician and stimulate discussions around feelings of dehumanization within therapy. Given the lack of practical examples and pilot data, these clinical implications warrant further empirical investigation and should be treated tentatively for now.

When recruiting participants outside of the NHS and members of the DiPS lived experience consultation group, study adverts specified positive symptoms of psychosis (hallucinations, delusions, and paranoia) and used the term “psychosis.” This meant that people who were involved in the development of items identified with the label of psychosis and experienced positive symptoms. Accordingly, items which included the word “psychosis” were endorsed. It is recognized that not all people seeking support for distressing sensory experiences will identify with the word “psychosis,” therefore may not feel this measure reflects their experiences. Furthermore, it is unclear if the measure fully captures dehumanization in relation to “negative” symptoms of psychosis, such as anhedonia, as people with these experiences were not specifically recruited when developing the measure.

During the Delphi study, consensus limits for inclusion (informed by previous Delphi studies in psychosis research^32^^,^^33^) were adjusted after Round 1 as no items met criteria for automatic inclusion. This might have been due to differences in language: items in the present studied were required to be rated “critical” for measuring self-dehumanization, whereas previous studies accepted ratings of “essential” or “important.” It may also reflect less alignment amongst stakeholders in the present study compared to previous research, and may therefore be considered a limitation.

Given the cross-sectional study design, it is impossible to determine whether self-dehumanization is a cause or consequence of distress in psychosis; longitudinal research is needed to understand how these experiences change over time. Correlational findings from the present study and qualitative work by O’Brien-Venus and colleagues^19^ suggest those who experience severe voice-hearing, paranoia, low self-esteem, diminished belonging, and reduced personal agency are at greater risk of self-dehumanization. Difficulties with self-esteem may manifest in different ways, for example with high levels of self-criticism being commonly found in people with depression^61^—it is possible that dehumanization-related low self-worth might be more common in psychosis, so the DiPS could be used in future research to explore this. Future research should clarify when and for whom the DiPS might be most useful, and assess its cross-cultural validity, measurement invariance, and sensitivity to clinical change. Self-dehumanization may be studied as a mechanism of change within therapy for psychosis, including mindfulness and compassion focused approaches. Psychological therapy for psychosis may also be developed to provide support for feelings of dehumanization.

The consideration of feelings of dehumanization is an important step forward in understanding distress in people who experience psychosis. The DiPS is a reliable, valid, and acceptable measure of self-dehumanization, which can be used within both research and therapeutic practice.

## Supplementary Material

SupplementaryMaterials_1_sbaf242

## Data Availability

Protocol for the DiPS validation study can be found here: https://osf.io/c6zfg/overview. Data and analysis code for the DiPS validation are deposited with the University of Bath Research Data Archive and can be accessed here: http://dx.doi.org/10.15125/BATH-01494

## Appendix

self-Dehumanization in Psychosis Scale (DiPS).

Please indicate how you have been feeling over the past week:

(Likert scale options 1-5. 1 = never, 2 = rarely, 3 = sometimes, 4 = often, 5 = always)

1. Psychosis prevents me from relating to other people.

2. I do not have control over my actions.

3. I am a valuable person*.

4. Distressing thoughts and voices make me feel like I am not human.

5. Psychosis has taken away who I really am.

6. I can’t trust myself to make good decisions.

7. I do not feel like a human being.

8. I cannot trust my mind.

9. I have a strong sense of who I am as a person*.

10. I do not belong in this society.

11. I see myself as less than human.

12. Psychosis has taken over who I am.

13. I am more than my experience of psychosis*.

*indicates reverse scoring.

Factors: Humanity = 4, 7, 10, 11; Identity = 1, 5, 12; Personhood = 3, 9, 13; Agency = 2, 6, 8.
