# Supplementary material for: Measuring feelings of dehumanization in people who experience psychosis: development and validation of the self-Dehumanization in Psychosis Scale (DiPS)
Source: Schizophr Bull. 2026 Mar 21;52(2):sbaf242. doi: 10.1093/schbul/sbaf242 (PMC13005108; doi:10.1093/schbul/sbaf242)
Supplement: SupplementaryMaterials_1_sbaf242 [file supplementarymaterials_1_sbaf242.docx]

**Supplementary materials**

**Item generation: Literature review**

***Search strategy***

APA PsychNet: "self dehuman*" OR "self infrahuman" = 22 papers

SCOPUS: "self dehuman*" OR "self infrahuman" = 21 papers

Web of science: "self dehuman*" OR "self infrahuman" = 24 papers

Pubmed: "self dehuman*" OR "self infrahuman" = 10 papers

Total = 77 papers

29 papers after removing duplicates

2 excluded for not identifying any theory or measure

3 excluded for not being a study (OSF pre reg with no data)

1 excluded as unpublished thesis

1 excluded as discussing dehumanisation (rather than self-dehumanisation)

Total = 22 papers

***Literature review: results***

A review of measures of self-dehumanisation identified 22 papers which either reference or develop their own measure of self-dehumanisation, in which there were eleven distinct measures of self-dehumanisation (see Table 1). After examination of the measures, it was decided that items from only six could be relevant to measuring self-dehumanisation in psychosis. These were Self-dehumanisation in People with Severe Alcohol Use Disorder Scale (with modifications) (Fontesse et al., 2021); Self-dehumanisation Scale (Bastian & Haslam, 2010); Mechanical Self-dehumanisation Scale (Sakalaki et al., 2017); Adaptation of Mind Attribution Scale (Kouchaki et al., 2018); Internalising Objectification and Self-dehumanisation Warmth and Competence Scale (Loughnan et al., 2017).

The remaining five scales were discarded, based on two being trait rating scales implicitly measuring self-dehumanisation (Low Human Nature Traits Scale (Sakalaki et al., 2016) and Humanness Traits Rating Scale (Bastian & Haslam, 2010)); two being emotion rating scales implicitly measuring self-infrahumanisation (McCleary-Gaddy & James, 2022; Renger et al., 2016) and animalistic self-dehumanisation (Animalistic Self-dehumanisation Scale (Sakalaki et al., 2017)) and one being a scale requiring participants to identify themselves on a Venn diagram of intersecting ‘animal’ and ‘machine’ circles (Adjusted Mechanistic Dehumanization of the Self Scale (Sakalaki et al., 2016)). These were deemed inappropriate for use in the DiPS, as they are either implicit measures, or a visual scale, rather than self-report statements. This means they would not fit the desired format for inclusion within the DiPS.

The six relevant measures contained a total of 13 (Fontesse et al., 2021), 12 (Bastian & Haslam, 2010), 14 (Sakalaki et al., 2017), 10 (Kouchaki et al., 2018), 11 (Loughnan et al., 2017) and 9 (Loughnan et al., 2017) items. The research team reviewed all items for appropriateness for inclusion in the DiPS. A total of 69 items were initially selected from the 5 scales. Of these, items were discarded for:

- Measuring a construct deemed as not self-dehumanisation (N=24)
- Measuring humanness traits, rather than the experience (N=18)
- Being worded in an ambiguous manner (N=13)
- Overlapping with or the same as another item (N=9)
- Containing stigmatising language or assumptions (N=2)

It was decided that only three items from all existing self-dehumanisation scales should be included in the deductive item shortlisting for the DiPS. These were all taken from the Bastian & Haslam (2010) Self-dehumanisation Scale. These items were ‘I feel mechanical and cold, like a robot’, ‘I feel less than human, like an animal’, and ‘I feel like an object, not a human’. These items were chosen because of their direct reference to one’s sense of humanness.

**Self-dehumanisation measurement**

***Table 1. A compendium of self-report measures quantifying self-dehumanisation***

| **Measure** | **N Items** | **Scale type** | **Underpinning theory** | **Target population** |
| --- | --- | --- | --- | --- |
| Self-Dehumanisation Measure (Bastian & Haslam, 2010) | 12 | 7-point Likert | Human nature & human uniqueness (Haslam, 2006) | General population |
| Self-Dehumanisation Traits measure (Bastian & Haslam, 2010) | 40 | 7-point Likert | Human nature & human uniqueness (Haslam, 2006) | General population |
| Measure of Self-Humanity (Bastian et al., 2013) | 8 | 7-point Likert | Human nature & human uniqueness (Haslam, 2006) | General population |
| Self-Dehumanization Scale in Patients with Severe Alcohol Use Disorder (Fontesse et al., 2021) | 13 | 7-point Likert | Human nature & human uniqueness (Haslam, 2006) | Patients with Severe Alcohol Use Disorder |
| Self-Dehumanisation Measure Adapted from Mind Attribution Scale (Kouchaki et al., 2018) | 10 | 7-point Likert | Emotion, intention, cognition (Kozak, 2006) | General population |
| Mechanistic Self-Dehumanisation Scale (Sakalaki et al., 2017) | 14 | 9-point Likert | Human nature (Haslam, 2006) | General population and healthcare workers |
| Adjusted Mechanistic Dehuamnisation of the Self Scale (AMDOSS) (Sakalaki et al., 2016) | 6 | Visual circles | Inclusion of Other in Self scale (Aron et al. 1992) and Human nature & human uniqueness (Haslam, 2006) | General population |
| Self-Infrahumanization Scale (McCleary-Gaddy & James, 2022; Renger et al., 2016) | 16 | 5-point Likert | Primary and secondary emotions (DeMoulin et al., 2004; Leyens et al., 2001) | African American women/students |
| Low Human Nature Traits Scale (Sakalaki et al., 2016) | 5 | 9-point Likert | Human nature (Haslam, 2006) | General population |
| Warmth and competence measure (Loughnan et al., 2017) | 9 | 5-point Likert | Stereotype content model (Fiske et al., 2002) | Women |
| Warmth, competence, and morality measure (Loughnan et al., 2017) | 9 | 5-point Likert | Stereotype content model (Fiske et al., 2002) | Women |

***Table 2. Cognitive interviews table of changes***

| **Interview Round** | **Interview feedback** | **Problem with item** | **Change made** |
| --- | --- | --- | --- |
| 1 | Statements 4, 14 & 15 are very similar | Repetition | Statement 4 has been removed |
| 1 | Wouldn’t know what ‘dehumanised’ means before participating in the study (statement 1) | Ambiguity | Statement 1 has been removed |
| 1 | Statement 2 is confusing and similar to statement 23; ‘thoughts and voices’ instead of psychosis could be more relatable | Ambiguity | Statement 2 changed to ‘distressing thoughts and voices make me feel like I am not human’ |
| 1 | Dislike wording of ‘my psychosis’ (statement 5) | Offensive | Statement 5 reworded to ‘my experience of psychosis’ to reflect its transient and changing nature |
| 1 | Unsure who you mean by ‘other people’ – some people accept me and others don’t | Ambiguity | Statement 6 changed to specify ‘I feel a sense of belong with people in my life’; statement 7 changed to ‘I do not belong in this society’ |
| 1 | Statement 9 is confusing and too wordy | Ambiguity | Statement 9 changed to ‘Psychosis prevents me from relating to other people’ |
| 1 | Statements 18 and 19 are the same in reverse | Repetition | Statement 18 changed to reflect ‘I do not have control over my actions and choices’, statement 19 changed to ‘I do not have control over life’ |
| 1 | Statements 21 and 22 are similar | Repetition | Added in ‘I can’t trust myself to make good decisions’ to better represent the totality of the domain (Trust in own credibility and reliability) |
| 2 | Statement 18 ‘I do not have control over my actions and choices’ is too wordy; actions and choices are the same | Ambiguity | Statement 18 reworded to ‘I do not have control over my actions’ |
| 2 | The word ‘influence’ in statement 19 throws me off | Ambiguity | Statement 19 reworded to ‘I do not have control over my life’ |
| 1&2 | Statement 22 - thoughts and feelings are different, and it is important to distinguish between them | Ambiguity | Statement 22 reworded to ‘I can’t trust my feelings’ |
| 1&3 | Statement 8 & 9 are very similar | Repetition | Statement 8 is removed |
| 1&3 | Statement 14 & 15 are very similar | Repetition | Statement 14 is removed |

**DiPS Validation**

***Figure 1. Parallel analysis graph for exploratory factor analysis***

**
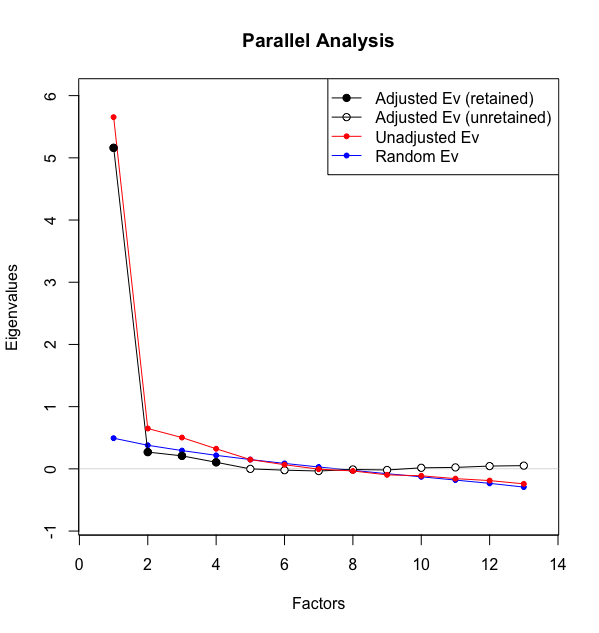
**

***Figure 2***

***Figure 3. Exploratory factor analysis loadings***

***
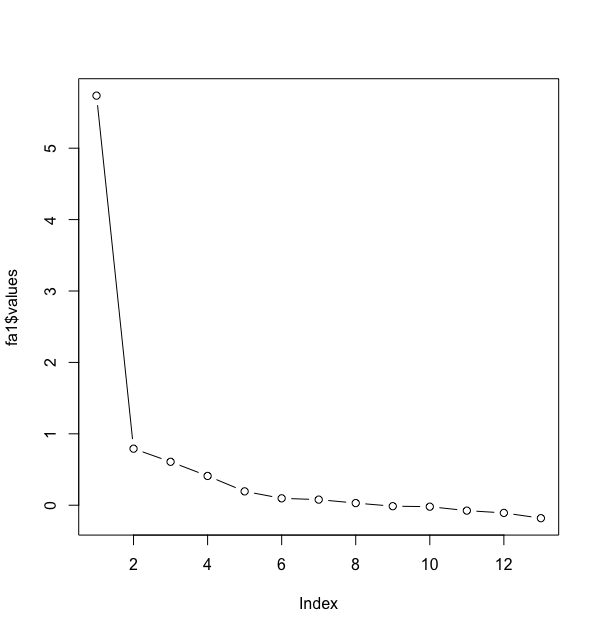
Figure 2. Scree plot for exploratory factor analysis***

***Table 3. Exploratory factor analysis rotated factor loadings for the DiPS***

| No. | Item | Communality | EFA 1 | EFA 2 | EFA 3 | EFA 4 |
| --- | --- | --- | --- | --- | --- | --- |

Factor 1: Humanity

| 7 | I do not feel like a human being | .69 | .84 |  |  |  |
| --- | --- | --- | --- | --- | --- | --- |
| 11 | I see myself as less than human | .77 | .84 |  |  |  |
| 4 | Distressing thoughts and voices make me feel like I am not human | .57 | .51 | .35 |  |  |
| 10 | I do not belong in this society | .58 | .46 |  |  | .31 |

Factor 2: Identity

| 5 | Psychosis has taken away who I really am | .74 |  | .85 |  |  |
| --- | --- | --- | --- | --- | --- | --- |
| 12 | Psychosis has taken over who I am | .74 |  | .82 |  |  |
| 1 | Psychosis prevents me from relating to other people | .47 |  | .37 |  |  |

Factor 3: Personhood

| 9 | I have a strong sense of who I am as a person | .77 |  |  | .89 |  |
| --- | --- | --- | --- | --- | --- | --- |
| 3 | I am a valuable person | .35 |  |  | .55 |  |
| 13 | I am more than my experience of psychosis | .43 |  |  | .44 |  |

Factor 4: Agency

| 6 | I can’t trust myself to make good decisions | .51 |  |  |  | .58 |
| --- | --- | --- | --- | --- | --- | --- |
| 8 | I can’t trust my mind | .57 |  |  |  | .48 |
| 2 | I have no control over my actions | .34 |  |  |  | .37 |

***Figure 3. Confirmatory factor analysis standardised factor loadings***

**
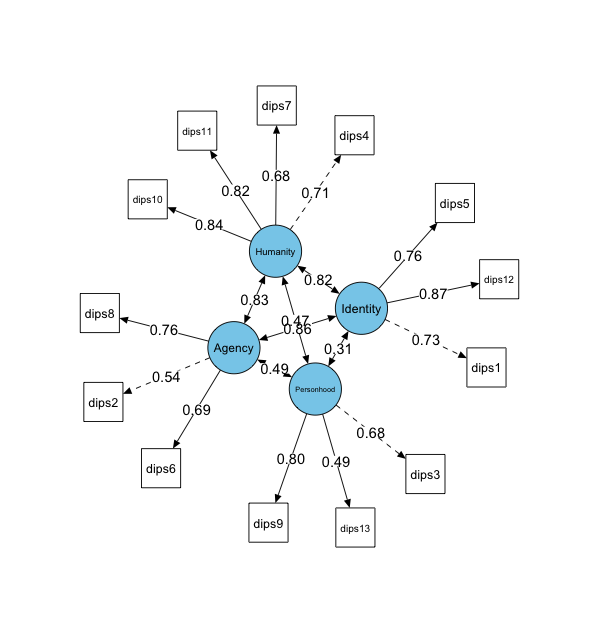
**

**DiPS 22-item**

Dehumanisation in Psychosis Scale (DiPS)

Please indicate how you have been feeling over the past week:

(Likert scale options 1-5. 1 = never, 2 = rarely, 3 = sometimes, 4 = often, 5 = always)

1. I can’t trust my feelings^1^
2. Psychosis prevents me from relating to other people
3. I have no private thoughts and feelings^1^
4. I do not have control over my actions
5. I feel fundamentally different to other people^1^
6. I am a valuable person*
7. Distressing thoughts and voices make me feel like I am not human
8. Psychosis has taken away who I really am
9. I can’t trust myself to make good decisions
10. I do not belong in this society
11. I am worth less than other people^1^
12. I can't trust my mind
13. I have a strong sense of who I am as a person*
14. I do not feel like a human being
15. I feel a sense of belonging with the people in my life*^1^
16. I do not have control over my life^1^
17. I see myself as less than human
18. I am less human than other people^1^
19. Psychosis makes me feel disconnected from the world^1^
20. I do not belong anywhere^1^
21. Psychosis has taken over who I am
22. I am more than my experience of psychosis*

*indicates reverse scoring

^1^removed for 13-item DiPS

**References**

Aron, A., Aron, E. N., & Smollan, D. (1992). Inclusion of Other in the Self Scale and the structure of interpersonal closeness. Journal of Personality and Social Psychology, 63(4), 596–612. [https://doi.org/10.1037/0022-3514.63.4.596](https://psycnet.apa.org/doi/10.1037/0022-3514.63.4.596)

Bastian, B., & Haslam, N. (2010). Excluded from humanity: The dehumanizing effects of social ostracism. *Journal of experimental social psychology*, *46*(1), 107-113. <https://doi.org/10.1016/j.jesp.2009.06.022>

Capozza, D., Colledani, D., & Falvo, R. (2021). Can secure attachment be related to prosocial behaviors through the mediation of humanizing self-perceptions? TPM-Testing, Psychometrics, Methodology in Applied Psychology, 28, 163-176.

Demoulin, S., Leyens, J. P., Paladino, M. P., Rodriguez‐Torres, R., Rodriguez‐Perez, A., & Dovidio, J. (2004). Dimensions of “uniquely” and “non‐uniquely” human emotions. *Cognition and emotion*, *18*(1), 71-96. <https://doi.org/10.1080/02699930244000444>

Fiske, S. T., Cuddy, A. J. C., Glick, P., & Xu, J. (2002). A model of (often mixed) stereotype content: Competence and warmth respectively follow from perceived status and competition. Journal of Personality and Social Psychology, 82(6), 878–902. [https://doi.org/10.1037/0022-3514.82.6.878](https://psycnet.apa.org/doi/10.1037/0022-3514.82.6.878)

Fontesse, S., Demoulin, S., Stinglhamber, F., de Timary, P., & Maurage, P. (2021). Metadehumanization and self-dehumanization are linked to reduced drinking refusal self-efficacy and increased anxiety and depression symptoms in patients with severe alcohol use disorder. *Psychologica Belgica*, *61*(1), 238. <https://doi.org/10.5334/pb.1058>

Haslam, N. (2006). Dehumanization: An integrative review. *Personality and social psychology review*, *10*(3), 252-264. <https://doi.org/10.1207/s15327957pspr1003_4>

Kouchaki, M., Dobson, K. S., Waytz, A., & Kteily, N. S. (2018). The link between self-dehumanization and immoral behavior. *Psychological science*, *29*(8), 1234-1246. <https://doi.org/10.1177/0956797618760784>

Kozak, M. N., Marsh, A. A., & Wegner, D. M. (2006). What do i think you're doing? Action identification and mind attribution. Journal of Personality and Social Psychology, 90(4), 543–555. [https://doi.org/10.1037/0022-3514.90.4.543](https://psycnet.apa.org/doi/10.1037/0022-3514.90.4.543)

Leyens, J. P., Rodriguez‐Perez, A., Rodriguez‐Torres, R., Gaunt, R., Paladino, M. P., Vaes, J., & Demoulin, S. (2001). Psychological essentialism and the differential attribution of uniquely human emotions to ingroups and outgroups. *European Journal of Social Psychology*, *31*(4), 395-411. <https://doi.org/10.1002/ejsp.50>

Loughnan, S., Baldissarri, C., Spaccatini, F., & Elder, L. (2017). Internalizing objectification: Objectified individuals see themselves as less warm, competent, moral, and human. *British Journal of Social Psychology*, *56*(2), 217-232. <https://doi.org/10.1111/bjso.12188>

McCleary-Gaddy, A. T., & James, D. (2024). Dehumanization, attitudes toward seeking professional psychological care, and mental health among African American women. Cultural Diversity & Ethnic Minority Psychology, 30(1), 166–176. [https://doi.org/10.1037/cdp0000554](https://psycnet.apa.org/doi/10.1037/cdp0000554)

Renger, D., Mommert, A., Renger, S., & Simon, B. (2016). When less equal is less human: Intragroup (dis) respect and the experience of being human. *The Journal of Social Psychology*, *156*(5), 553-563. <https://doi.org/10.1080/00224545.2015.1135865>

Ridge, D., Bullock, L., Causer, H., Fisher, T., Hider, S., Kingstone, T., ... & Southam, J. (2023). ‘Imposter participants’ in online qualitative research, a new and increasing threat to data integrity?. *Health expectations: an international journal of public participation in health care and health policy*, *26*(3), 941. <https://doi.org/10.1111/hex.13724>

Sakalaki, M., Richardson, C., & Fousiani, K. (2016). Self-dehumanizing as an effect of enduring dispositions poor in humanness. *Hellenic Journal of Psychology*, *13*(2), 104-115.

Sakalaki, M., Richardson, C., & Fousiani, K. (2017). Is suffering less human? Distressing situations' effects on dehumanizing the self and others. *Hellenic Journal of Psychology*, *14*(1), 39-63.
